# Supplementary material for: Asynchronous magnetic resonance elastography: Shear wave speed reconstruction using noise correlation of incoherent waves
Source: Magn Reson Med. 2022 Oct 27;89(3):990–1001. doi: 10.1002/mrm.29502 (PMC9792433; doi:10.1002/mrm.29502)
Supplement: Supplementary file 1 — DATA S1 MATLAB code used to generate the numerical simulations of Figure 1 [file MRM-89-990-s002.zip › k-Wave/helpfiles/resize.html]

resize :: Functions (k-Wave)


# resize

Resize a matrix.

## Syntax

```
mat_rs = resize(mat, new_size)
mat_rs = resize(mat, new_size, interp_mode)
```

## Description

`resize` resamples a matrix to a given size using `interp1` (1D), `interp2` (2D), or `interp3` (3D).

## Inputs

|  |  |
| --- | --- |
| `mat` | matrix to resize |
| `new_size` | desired matrix size in elements given by [N] in 1D (a single number is used for both row and column vectors), [Nx, Ny] in 2D and [Nx, Ny, Nz] in 3D. |

## Optional Inputs

|  |  |
| --- | --- |
| `interp` | interpolation mode used by `interp1`, `interp2`, and `interp3` (default = `'*linear'`) |

## Outputs

|  |  |
| --- | --- |
| `mat_rs` | resized matrix |

## Examples

- Loading External Image Maps
- 2D Time Reversal For A Circular Sensor
- Optimising k-Wave Performance

## See Also

`interp1`, `interp2`, `interp3`
